# The Book World of Medicine and Science

**Published:** 1898-04-02

**Authors:** 


### The Book World of Medicine and Science.

Diseases of the Nervous System: A Handbook for Students and Practitioners. By Charles E. Beevor, M.D.Lond., F.R.C.P., Physician to the National Hospital for the Paralysed and Epileptic, &c. (London: H. K. Lewis. 1898. Pp. 436. Price 10s. 6d.)

This is a good clinical guide to diseases of the nervous system. It is not very profound; perhaps some might say that in its phraseology it is not strikingly modern; but in its description of facts it is accurate, and in regard to treatment it is safe. We can have no doubt that the method of case taking recommended by Dr. Beevor is far the best plan to adopt. Half the mistakes made in general practice about diseases of the nervous system arise from going by first impressions and fitting the line of investigation to the assumed locality of the disease; and nothing can eliminate this source of error more effectually than the complete examination advised by Dr. Beevor, according to which each system—*i.e.*, motor, sensory, &c.—is taken separately and described throughout. The book commences with a good account of the anatomy and physiology of the nervous system. This is followed by a description of the method of taking a case and conducting an examination. The various diseases and their local manifestation are then described, and finally some useful tables are given showing the topographical relations of the various nerves and some plates illustrating their distribution. It seems to us that this book will be very useful for the purpose for which it is intended, viz., as a practical handbook for students and practitioners. It is, however, with a certain amount of surprise that we note the absence of all reference to certain recent views on the intimate construction of the nervous system. Neither the word "neuron" nor "dendron" appears in the index, nor, so far as we have seen, in the book itself. The book is very well balanced, no excessive prominence being given to any particular disease, and some useful illustrations are introduced, many of them being original.

Exercise for Health. By H. H. Hulbert, B.A., M.R.C.S., and Luis J. Phelan. (London: The Whiteley Exerciser (Limited) and A. C. Pearson.) 1898. Pp. 260. Diagrams. Price 3s. 6d. net.

This book is written in three parts: the first is a series of essays on food, training, treatment of deformities, and other topics; the second, a popular account of some anatomical facts; the third, a series of charts illustrating the use of the Whiteley Home Exerciser. In the first part—notably in the essay on "Health in the City," by the late Dr. Mannington Caffyn—are some excellent and judicious remarks of general interest. But the purpose of the book obviously is the exaltation of Mr. Luis Phelan's use of the Whiteley Home Exerciser as "the only ideal form of daily exerciser." We are not enamoured of these home exercisers. They are all more or less an arrangement of pulleys and indiarubber bands, and no doubt may serve to develop certain muscles or sets of muscles, and may even tend to correct deformities due to muscular weakness. But there is no doubt that the chief therapeutic end of exercise can only be attained by exercise in the fresh air and sunshine. And there is a very real danger lest exercise, undertaken under conditions which do not allow of rapid excretion and free oxidation, directly add to the "waste products" of an already overcharged organism. An hour's walk across a public park will do a tired clerk more good than any amount of gymnastics with indiarubber bands in a gas-heated living room, and a few extra inches of chest girth are dearly purchased by the forced production of emphysema. There are not a few blunders in this book. The authors refer to Sir J. Crichton Browne as "Dr. Creighton Brown," and accredit Mr. C. Heath with the titular distinction they deny to his medical confrère.

The Medical Examination for Life Assurance. By F. de Havilland Hall, M.D., F.R.C.P. Price 2s. 6d. net. Pp. 69. (Bristol: J. Wright and Co. 1898.)

This little book is a résumé of the facts it is essential for a medical examiner to bear in mind when rating a candidate for life assurance. As such it is heartily to be commended. Dr. de Havilland Hall's rulings—as is perhaps only natural—turn the scale rather often in favour of the assurance offices, but no other exception can be taken to them. We confess to a feeling of disappointment at the reserve with which so distinguished a physician as Dr. Hall treats the question of functional albuminuria. Dr. Hall deems it at present safer not to recommend candidates who exhibit albumen in their urine. But by albumen Dr. Hall means albumen coagulable by boiling and dilute acids. Therefore many cases of "functional" albuminuria must be regarded as outside Dr. Hall's ruling. There are, at the end of the book, some excellent remarks on the choice of an assurance office, and a valuable comparative table. We would welcome, in a subsequent edition, some expression of opinion from Dr. Hall on the relative importance—from the assurance offices' point of view—of the early signs and symptoms of disease.

Manual of Static Electricity. By S. H. Monell, M.D. Illustrated. Pp. 622. (Second Edition. New York, 1897. William Bensley Harrison.)

This elaborate treatise is devoted to an exposition of the use of static electricity in medicine. The author—who is an enthusiast—believes that the disrepute into which static electricity has fallen with physicians is due to the defects of imperfect apparatus. Dr. Monell believes that in the Holtz machine perfection has been reached, and that static electricity will now resume its proper place as a therapeutic agent. Be that as it may, we are glad to note that Dr. Monell recognises natural limitations to the use of his methods. He does not claim for static electricity any great influence on organic disease, and he recognises that its usefulness lies chiefly in the relief of pain, and the cure of functional maladies. That being so, of course static electricity must take its place by the side of other remedies which are remedies by moral effect. Even so, as Dr. Monell shrewdly says, is it not better to cure a neurotic woman by a few applications of electricity than to put her husband to the expense of travel and a fruitless voyage to Europe? Dr. Monell has, however, found uses other than the cure of neuroses to which the Holtz machine may be put. For months past Dr. Monell says he has satisfactorily used this machine as a substitute for the coil in X-ray work. The author's work in this direction must be judged by experts, but it is detailed with great care and an evident command of technique. "Static Electricity" is an interesting book, for, as we have said, the author is an enthusiast and speaks from his own experience. Its interest is enhanced by the idiomatic piquancy of many of the expressions used, so welcome after the stilted pomposity of much "professional" English. For two phrases in particular we are grateful to Dr. Monell: his description of a well-known class of patients as "gynæcological wrecks," and his definition of them as "women with an unimpaired capacity for getting sick and feeling miserable."

